# Perceptual Grouping of Closed Contours Is Disrupted by the Interpretation of the Scene Layout

**DOI:** 10.3389/fnbeh.2017.00164

**Published:** 2017-09-08

**Authors:** Junjun Zhang, Chaoyang Wan, Zhenlan Jin, Ling Li

**Affiliations:** Key Laboratory for NeuroInformation of Ministry of Education, High-Field Magnetic Resonance Brain Imaging Key Laboratory of Sichuan Province, Center for Information in Medicine, School of Life Science and Technology, University of Electronic Science and Technology of China Chengdu, China

**Keywords:** closure grouping, scene interpretation, perceptual organization, object-based attention, visual search, stereopsis

## Abstract

Closure is one of the grouping principles in perceptual organization. Studies have shown that closure can be affected by several factors, specifically by low-level image features. However, the effects of high-level non-image factors on grouping by closure are unknown. In two experiments we investigated how closure is affected when depth information is introduced to the 2D closed contours, whilst the other 2D features remain intact. The first experiment showed that the grouping of closed contours was disrupted by the manipulation in 3D layout. The second experiment showed that thus disruption resulted in the impairment of searching efficiency. These findings suggest that closure is not only determined by the image features, but also affected by the interpretation of the contextual scene layout.

## Introduction

Closure, one of the basic principles in Gestalt psychology, is considered to group contours into a perceptual whole (Brooks, 2015). Even fragments of a closed contour can be perceived as closed (Elder and Zucker, 1994). A number of studies have shown that the detection (Kovács and Julesz, 1993; Mathes and Fahle, 2007; Zhang et al., 2009; Gerhardstein et al., 2012) and discrimination (Elder and Zucker, 1993, 1994; Saarinen and Levi, 1999; Kanbe, 2013) of closed contours is facilitated compared to open contours. These studies investigated different 2D image features of closure. However, recently it has been argued that in addition to the features of an image, perceptual organization may also be affected by other factors (Wagemans et al., 2012; Brooks, 2015), such as probability (Beck and Palmer, 2002) and learning (Vickery and Jiang, 2009). This raises the question of whether closure depends solely on image features, or whether it is also affected by other non-image factors.

Electrophysiological (Doniger et al., 2000; Sehatpour et al., 2008) and neuroimaging (Altmann et al., 2003) studies found that closure activates the lateral occipital complex (LOC), which plays an important role in visual object recognition (Sehatpour et al., 2006). TMS studies showed disruption of closure was induced by applying TMS stimuli over extrastriate cortex (Brighina et al., 2003) and dorsal visual pathway (Amiaz et al., 2016). These studies also suggested that perceptual closure may be affected by top-down modulations.

Why does closure facilitate grouping? There has been evidence to suggest that a closed contour acts as a perceptual bridge between a 1D contour and a 2D shape (Elder, 2015). The closed contour forms the boundaries of a visual object and thus facilitates performance. In natural scenes, different parts of the contour are often occluded by other objects, leaving only fragments of the contour visible. Even under these circumstances, fragments of a closed contour can still be perceived as boundaries of a visual object so that these can also be detected and discriminated faster than open contours. It is important to note that fragments of a closed contour can still form the boundaries of an object, since the missing gaps are assumed to be “non visible” rather than “nonexistent”. On the other hand, when the gaps are known to be “nonexistent”, the contours are not being able to form the boundary of an object. Thus, the question arises of whether grouping is disrupted when closure is perceived as a non-closure.

The concept of closure in Gestalt psychology refers to “geometrical closure”, as it only focuses on the geometry of the closed lines. However, “geometrical closure” does not guarantee the formation of the perceptual grouping of closed contours. Thus, only a “perceptual closure” can form the boundary of a visual object. The key point is that the interpretation of the gaps where the contour discontinues determines the closure of the contours.

In the current study, we hypothesized that the “perceptual closure” determines the formation of a visual object rather than the “geometrical closure”, and that this would lead to a facilitation of the performance. To test this hypothesis, we used a stereogram to manipulate the depth order in the scene layout. Such manipulations have been shown to alter the perception of subjective contours (Gillam and Nakayama, 2002) and surface grouping (Nakayama, 1996).

Two experiments were carried out. In the first experiment, we tested whether disruption of “perceptual closure” impairs the formation of an object. A modified object-based attention paradigm was utilized. The rationale was that if the closed contours are grouped within the boundary of an object, attention will also be focused within the boundaries of this object. Thus, a target that appears within the object will be detected faster. On the other hand, if the closed contours are not grouped within the boundaries of an object, detection of a target will not be facilitated. In the second experiment, we further examined whether the formation of the object affects the efficiency in a visual search task. The rationale was that if the grouping of the closed contours of the searched items is disrupted, the formation of these items will also be disrupted, and in turn the searching efficiency would be impaired.

## Experiment 1

**Figure 1:**
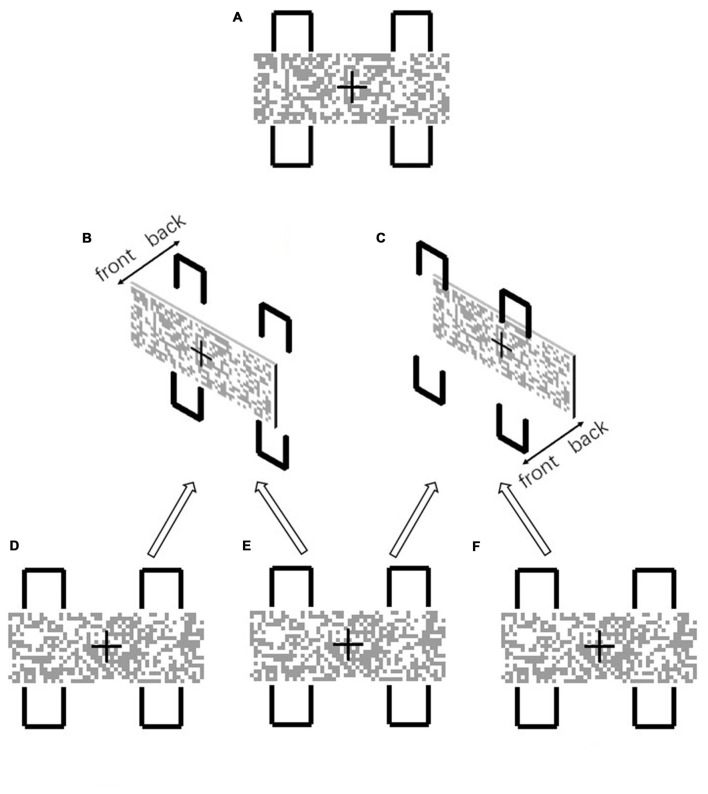
**(A)** In the 2D configuration, the two left and two right contours formed two visual objects. Detection of the same-object target was facilitated due to object-based attention, since the mosaic bar seems to occlude the contours and the gaps of the contours are perceived as “non visible” **(B)**. When the mosaic bar was placed behind the contours **(C)**, the gaps were no longer perceived as occluded and were perceived as “non existent”. The stereogram presentation **(B)** was formed from the uncrossed fusion of **(D,E)**. **(C)** was formed from the uncrossed fusion of **(E,F)**.

Experiment 1 examined whether the manipulation of depth information can disrupt object formation when the geometrical contour closure is identical. We utilized the two-rectangle paradigm (Shomstein, 2012) for this object-based attentional experiment. As shown in Figure 1A, a mosaic bar and four contours were presented in 2D. The two left and two right contours formed the fragmented boundaries of two different objects, when the mosaic bar appeared in front of the contours (Figure 1B). We examined how this effect was modulated when the depth order was altered, so that the mosaic bar was placed behind the four contours. As illustrated in Figure 1C, the geometrical closure feature of the contours remained the same, however the 3D scene layout appeared as though the discontinuity of the contours was not due to an occlusion of the object. In other words, we examined whether the change in depth order disrupted the grouping of the contours into visual objects.

### Materials and Methods

#### Subjects

Twenty-four college students (10 females, age 19–26 years, mean age 21.8 years) participated in the first experiment. All the participants were right-handed and had normal or correct-to-normal vision. This study was carried out in accordance with the recommendations of “the Guideline for Human Behavior Studies, the Institutional Review Board of UESTC MRI Research Center for Brain Research” with written informed consent from all subjects. All subjects gave written informed consent in accordance with the Declaration of Helsinki. The protocol was approved by the Institutional Review Board of UESTC MRI Research Center for Brain Research.

#### Apparatus

A 23″ LED monitor with a resolution of 1920 * 1080 was used in the experiment. A four-mirror stereoscope was placed in front of the monitor with a distance of around 57 cm. A chin rest was placed in front of the stereoscope. The height of the chin rest was adjustable so that the participants’ eyes were at the same height as the stereoscope. A black box covered the area around the monitor and the stereoscope, to ensure that participants could only view the monitor through the stereoscope.

#### Stimuli

**Figure 2:**
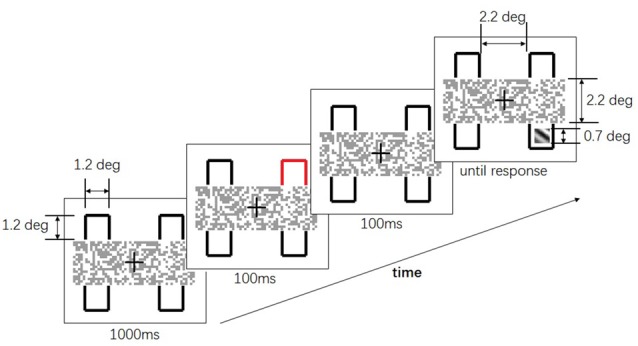
The time course of a single trial in Experiment 1. After 1000 ms, one of the four contours flashed for 100 ms. After another 100 ms interval, a squared Gabor patch showed up at two possible positions: either horizontally aligned to or vertically aligned to the cued contour. Participants were asked to judge the orientation of the Gabor patch as soon as possible.

In the experiment, participants were asked to maintain their fixation towards the center of the display during the whole trial. In each block, one of the two layouts of the two-rectangles was presented throughout. One of the layouts was as in Figure 1B and another was as in Figure 1C. The layout in Figure 1B was referred to as the “occluded” condition, uncross-fused from Figures 1D,E, since the contours were occluded by the mosaic bar. The layout in Figure 1C was referred to as the “non-occluded” condition, uncross-fused from Figures 1E,F. The whole layout subtended 4.5° (w) * 5.5° (h) and a viewing distance of 57 cm. The time course of a single trial was depicted in Figure 2. At the beginning of each trial, the layout was presented for 1000 ms. Afterward, one of the four contours changed color to red for 100 ms, serving as a cue. After a 100 ms interstimulus interval, a squared Gabor patch with a size of 0.7° * 0.7° was presented at two possible positions. One position was within the contour, which was horizontally aligned with the cued contour (referred to as “different-object” condition), another position was within the contour, which was vertically aligned with the cued contour (referred to as “same-object” condition). The Gabor bar was oriented either 45° left or 45° right. Participants were asked to judge the orientation of the Gabor bar by pressing one of the two keys designated as quickly as possible. The Gabor bar disappeared right after the key was pressed. Only the reaction times for the correct responses were subject to further analysis.

#### Design

The independent variables were the depth order (“occluded” and “non-occluded”) and the target position (“same-object” and “different-object”). Thus, there were four conditions: two depth orders * two target positions. Each condition was replicated 32 times. There were four blocks of 32 trials in the experiment. In each block, only one depth order was presented. The position of the cued contour was randomized across trials. The block order of the depth order was counterbalanced between subjects. In each block, the trials were randomized across participants.

### Results

**Figure 3:**
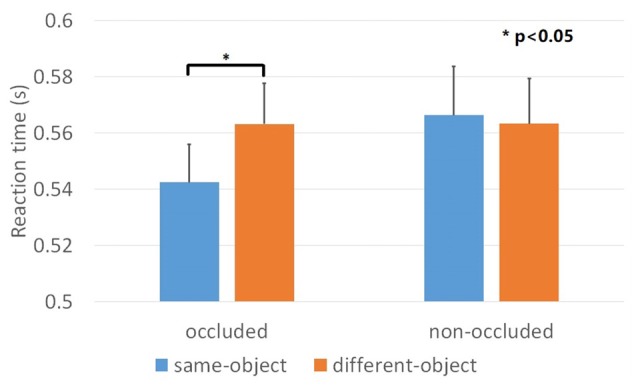
Results of Experiment 1. Mean reaction time across the four conditions. The object-based attention effect was observed when the mosaic bar was placed in front of the contours (occluded condition). Such effect was not observed when the mosaic bar was placed behind the contours (non-occluded condition). Error bars represented standard errors.

We evaluated and compared two independent variables: (1) depth order (i.e., “occluded” and “non-occluded”); and (2) target position (i.e., “same-object” and “different-object”). The dependent variable was the mean reaction time of the correct responses. The percentages of corrected responses for all conditions were above 95% and there were no main effects nor interactions. The result of one-sample Kolmogorov-Smirnov showed that the data for each condition was normally distributed. We used a repeated ANOVA for the comparisons and found a significant main effect for target position, *F*_(1,23)_ = 6.17, *p* < 0.05, $\eta_{\text{p}}^{2}$ = 0.211 and a significant depth order × target position interaction, *F*_(1,23)_ = 6.57, *p* < 0.05, $\eta_{\text{p}}^{2}$ = 0.222 (Figure 3). When the contours were occluded, the reaction time for the “same-object” condition was significantly decreased compared to the “different-object” condition, *F*_(1,23)_ = 15.5, *p* < 0.01, $\eta_{\text{p}}^{2}$ = 0.403, indicating an object-based attention effect. However, such an effect was not observed for the “non-occluded” condition. The reaction times for the “same-object” and “different-object” conditions were 566 ms and 563 ms, respectively, showing no significant differences, *F*_(1,23)_ = 0.082, *p* = 0.777, $\eta_{\text{p}}^{2}$ = 0.004. This result suggests that the depth order disrupted the grouping of the geometrical closed contours. Thus, when the gaps of the contours were clearly shown to be non-occluded, the contour fragments did not group together as a whole visual object any more.

## Experiment 2

**Figure 4:**
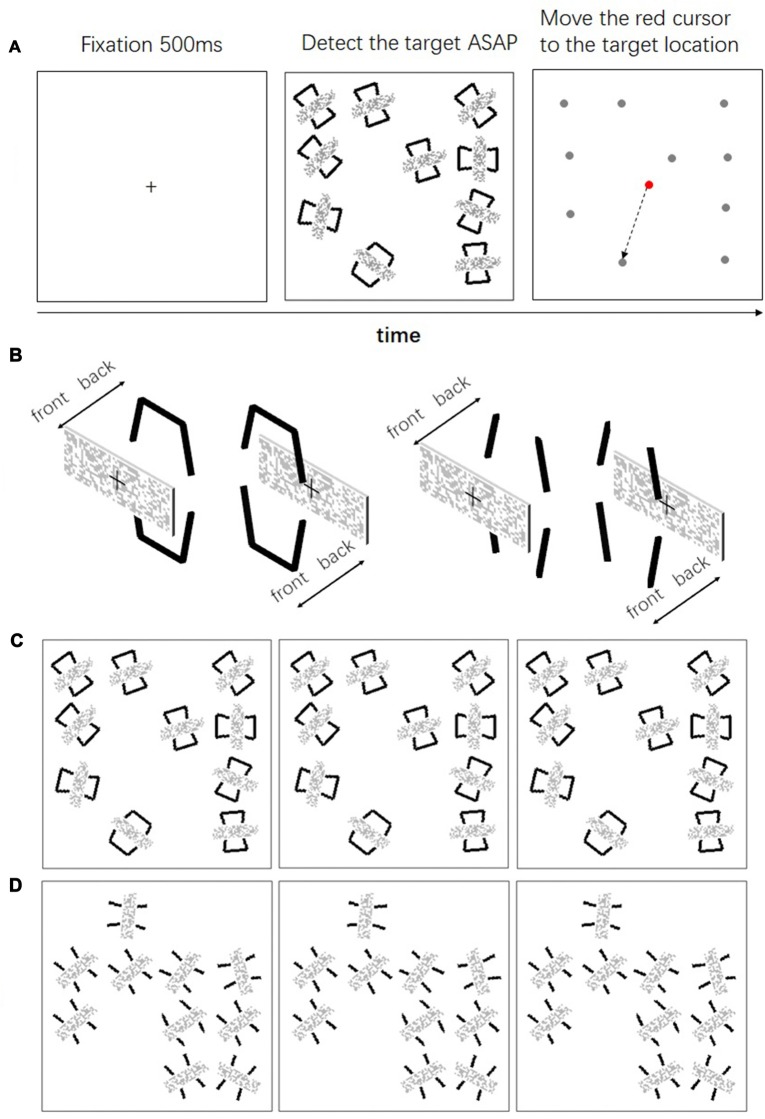
**(A)** The time course of a single trial in Experiment 2; **(B)** depth manipulation of the contours and the mosaic bars; **(C)** the left pair (the left and the middle panels) is the uncrossed fusion of searching occluded closed contours, the right pair (the middle and the right panels) is the uncrossed fusion of searching occluding closed contours; **(D)** the left pair is the uncrossed fusion of searching occluded open contours, the right pair is the uncrossed fusion of searching occluding open contours.

Experiment 2 examined whether the performance was facilitated when the geometrical closed contours did not form a visual object any longer. To this end we used a visual search task similar to that of Elder and Zucker (1993). Here we manipulated both the geometrical closure and the depth order of the items in the search task. Geometrical closed and open contours were used to form the search items. A mosaic bar was placed either in front of or behind these contours (same as in Experiment 1). We examined the searching efficiency in these four conditions (Figure 4B).

### Materials and Methods

#### Subjects and Apparatus

Twenty-four college students (12 females, age 19–28 years, mean age 22.5 years) participated in the second experiment. None of them have participated in the first experiment. All the participants were right-handed and had normal or correct-to-normal vision. This study was carried out in accordance with the recommendations of “the Guideline for Human Behavior Studies, the Institutional Review Board of UESTC MRI Research Center for Brain Research” with written informed consent from all subjects. All subjects gave written informed consent in accordance with the Declaration of Helsinki. The protocol was approved by the Institutional Review Board of UESTC MRI Research Center for Brain Research. The apparatus was the same as in the first experiment.

#### Stimuli

In each trial, a square was presented at the center of the monitor. The square subtended 8° (w) * 8° (h) with a viewing distance of 57 cm. The search area was within this square and was divided into 16 possible locations, each of which subtended 2° * 2° (Figure 4A). In each trial, 10 locations were selected randomly as the position for the searching items. Each item subtended 1° * 1.5°. The orientation of each item was randomized. In order to eliminate the alignment of the items, each item was randomly placed within a 0.2° horizontal and vertical distance from the center of the location. At the beginning of each trial, a fixation cross was presented at the center of the display for 0.5 s, 10 searching items were presented thereafter. Among the 10 items, only one of the items was the target and the others were distractors. Participants were asked to click the left mouse button as soon as possible after they detected the target. The search items were replaced by a dot (0.2° * 0.2°) immediately after the response. After that, participants were asked to move a cursor with the mouse to the location where the target was presented. Only the reaction times for the correct trials were subject to further analysis.

#### Design

The independent variables were the depth order (Figure 4B, the same “occluded” and “non-occluded” conditions as in Experiment 1) and the closure contour (Figures 4C,D, open and closed). Thus, there were four conditions: two depth orders * two closure contours. Each condition was replicated 32 times. There were four blocks of 32 trials in the experiment. In each block, only one depth order was presented. The order of the depth order was counterbalanced between subjects.

### Results

**Figure 5:**
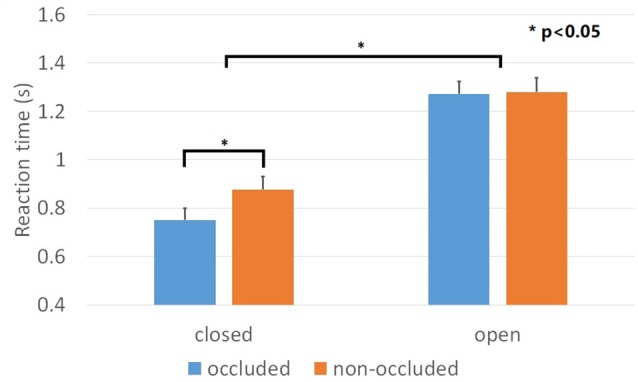
Results of Experiment 2. Mean reaction time across closed and open contours when these are either occluded or non-occluded. Discriminating among the items of closed contours was faster than that of open contours. However, the manipulation of the depth order only affected closed contours but not open contours. Error bars represented standard errors.

The percentages of corrected responses for all conditions were above 97% and there were no main effects nor interactions. The result of one-sample Kolmogorov-Smirnov showed that the data for each condition was normally distributed. We evaluated and compared the mean reaction time of the correct responses using a repeated ANOVA with the geometrical closure and the depth order as factors. We found main effects for geometrical closure, *F*_(1,23)_ = 138.6, *p* < 0.001, $\eta_{\text{p}}^{2}$ = 0.858, depth order, *F*_(1,23)_ = 9.036, *p* < 0.01, $\eta_{\text{p}}^{2}$ = 0.282 and a significant geometrical closure × depth order interaction, *F*_(1,23)_ = 6.023, *p* < 0.05, $\eta_{\text{p}}^{2}$ = 0.208. Geometrical closure facilitated searching efficiency for the “occluded” condition, *F*_(1,23)_ = 135.0, *p* < 0.001, $\eta_{\text{p}}^{2}$ = 0.854 and the “non-occluded” condition, *F*_(1,23)_ = 72.5, *p* < 0.001, $\eta_{\text{p}}^{2}$ = 0.759 of the contours (Figure 5). Regarding the depth order manipulation, the searching efficiency for the geometrical closed contour decreased, *F*_(1,23)_ = 17.7, *p* < 0.001, $\eta_{\text{p}}^{2}$ = 0.434. The reaction time for the occluded and occluding conditions were 751 ms and 877 ms, respectively. However, the searching efficiency for the geometrical open contour remained intact, *F*_(1,23)_ = 0.039, *p* = 0.845, $\eta_{\text{p}}^{2}$ = 0.002. The reaction time for the occluded and occluding conditions were 1272 ms and 1279 ms, respectively.

In Experiment 2, the mosaic bar disrupted the organization of the contours. Not only was the formation of the perceptual closure disrupted, but also the continuity of both the closed and open contours. Adequate contour continuation is considered to be an important grouping principle (Brooks, 2015). However, in the current study manipulating the depth order did not impair the searching efficiency of the open contours, suggesting that the disruption of the contour continuity did not affect performance. This suggests that the decline in the searching efficiency of the items with closed contours was due to closure rather than contour continuity. In the conditions where the objects were occluded, items with closed contours were still easier to discriminate compared to those with open contours. This suggests that geometrical closure plays an important role in grouping effects.

## Discussion

The present study directly demonstrated for the first time that the formation of a visual object, which is defined by fragmented closed contours, depends on the “perceptual closure”, rather than the “geometrical closure” of the contours. The definition of a visual object has always been one of the central questions in perception. Studies have proposed that an object is not defined only by the image features but also by perceptual factors (Scholl, 2001; Feldman, 2003). However, little empirical evidence has directly addressed this proposal. Most of the previous studies focused on how image-based features affect grouping of objects (Wagemans et al., 2012). A few non-image factors that affect grouping have also been studied (Brooks, 2015). However, how the understanding of a scene can affect the grouping of contours has not been studied. Our findings suggest that grouping of contours depend, on the level of the scene understanding and not on geometrical grouping cues.

In Experiment 1, our findings showed that visual grouping of the contours depended on the depth order of the contours with respect to the mosaic bar. From a 2D perspective, the mosaic bar was always assumed to be in front of the contours, so that the layout in Figure 1A was the same as that in Figure 1B. Thus, the grouping of the contours required an amodal completion. In other words, the perceived unity was partly occluded by other objects. According to Anderson et al. (2002) amodal completion only requires occlusion, thus the contours were completed by the boundaries of the two objects. Participants assumed that the contours existed behind the mosaic bar but these were in fact unseen. As a result, an object-based attentional effect was observed (Figure 1B). These findings are in accordance with other 2D studies on object-based attention and occlusion (Behrmann et al., 1998; Pratt and Sekuler, 2001). Furthermore, neurophysiological studies have shown that neurons in higher areas of the visual cortex are sensitive to amodal completion (Tso and Roe, 1995; Weigelt et al., 2007), suggesting that amodal completion not only depends on simple image features.

On the other hand, when the mosaic bar was placed behind the gaps, as in Figure 1C, no occlusion occurred. Here the completion of the boundaries required a modal completion, i.e., completion of the gaps where there is no image contrast. Thus, when the mosaic bar was placed behind the gaps the luminance relationship was disrupted, and this in turn disrupted the modal completion (Anderson et al., 2002). In other words, the participants knew that there was no object in front of the gaps, so that no contours were perceived. Thus, the geometrical closed fragmented contours were not perceived as a coherent boundary and the formation of the visual object was disrupted. As a result, the object-based attentional effect was not observed in the layout that is displayed in Figure 1C.

In the current study, Experiment 1 demonstrated that the grouping of the boundaries of an object depended on the interpretation of the 3D layout. In Experiment 2, we further showed that the manipulation of the object formation affected the facilitation of closure grouping. Previous studies proposed that the facilitation of closure grouping is due to the formation of a visual object (Elder, 2015), however, there was no direct evidence for this. Here we show for the first time, that the disruption of an object formation impairs the searching efficiency.

Nakayama et al. (1989) and Nakayama (1996) have already shown that the depth order affects perceptual organization. In their studies, the occluded and occluding objects always shared boarders. The ownership of the shared borders plays an important role in the perceptual integration. In a more recent review (von der Heydt, 2015), “boarder ownership” was also emphasized to be important in the formation of so named “proto-object”, which described an early guess of the object formation in a scene. In this study, we deliberately avoided the “boarder ownership”. In the two experiments reported, the color of the mosaic bar was set to gray and the color of the contours was set to black to make a clear distinction between them. Thus there is not a single border that can be perceived as shared by the contours and the mosaic bar. In this case, when the contours were placed in front of the mosaic bar, there are no “extrinsic” borders (Nakayama et al., 1989) assigned to the contours. However, when the contours were placed at the back of the mosaic bar, it’s still possible that the edge of the mosaic bar can be seen as the “extrinsic” border to the contours. Thus the contours above and below the mosaic bar tended to link with each other to form an object. Hence, we argue that the disruption of object formation does not necessarily depends on the boarder ownership, but depends on a more general interpretation of the 3D scene layout. Another important point made by the previous studies (Nakayama et al., 1989; von der Heydt, 2015) was that this integration in 3D occurs at an early stage in perceptual organization. Such an organization forms the “proto-object” in order to provide a structure for selective-attention. Our experiments supported this suggestion by showing that the depth manipulation affected both the object-based attention (Experiment 1) and the visual search (Experiment 2) tasks.

Our findings extend the hypothesis of the 3D contour interpolation proposed by Kellman et al. (2005), which proposed that object perception in 3D requires contour interpolation in 3D. The proposition was that a 3D interpolation of a pair of contours occurs only when the pair is relatable. Two relatable contours require three constraints: smoothness, monotonicity and a 90° limit. In the current study we showed that even when the contours are relatable in 3D geometry, the contour interpolation may still not occur due to the interpretation of the scene. When the contours were placed behind the mosaic bar, they were perceived as occluded and as the outline of a single object, which is in line with the aforementioned study (Kellman et al., 2005). However, when the contours were placed in front of the mosaic bar the modal completion did not happen. In this case, even though the 3D geometry was relatable, the contours did not form the outline of a single object.

## Author Contributions

JZ and LL designed research; JZ and CW performed research; JZ analyzed data; JZ, ZJ and LL wrote the article.

## Conflict of Interest Statement

The authors declare that the research was conducted in the absence of any commercial or financial relationships that could be construed as a potential conflict of interest.
